# Supplementary figures and images for: Effects of plasminogen activator inhibitor-1 deficiency on bone disorders and sarcopenia caused by adenine-induced renal dysfunction in mice
Source: PLoS One. 2024 Oct 10;19(10):e0311902. doi: 10.1371/journal.pone.0311902 (PMC11469609; doi:10.1371/journal.pone.0311902)

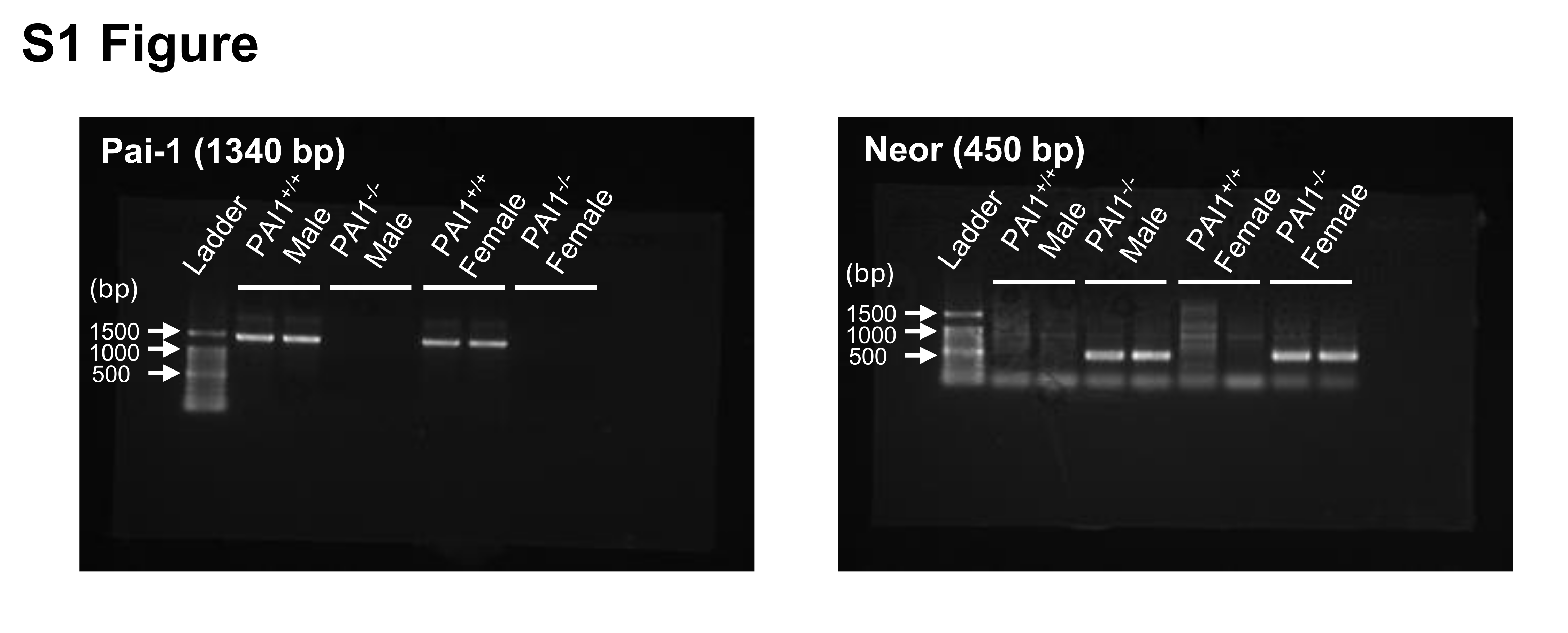

Supplement: S1 Fig — (Left) Genotyping by PCR was performed to identify PAI-1-/- mice with Pai-1 primer. The absence of a PCR product indicates the genomic knockout of Pai-1. The band shows a PCR product of 1340 bp. (Right) Genotyping by PCR was performed to confirm PAI-1-/- mice with Neor primer. The presence of a PCR product indicates the genomic knock in of Neor in the process of gene editing. The band shows a PCR product of 450 bp. The lower bands represent unspecific primer dimers. (TIF) [file pone.0311902.s001.tif]

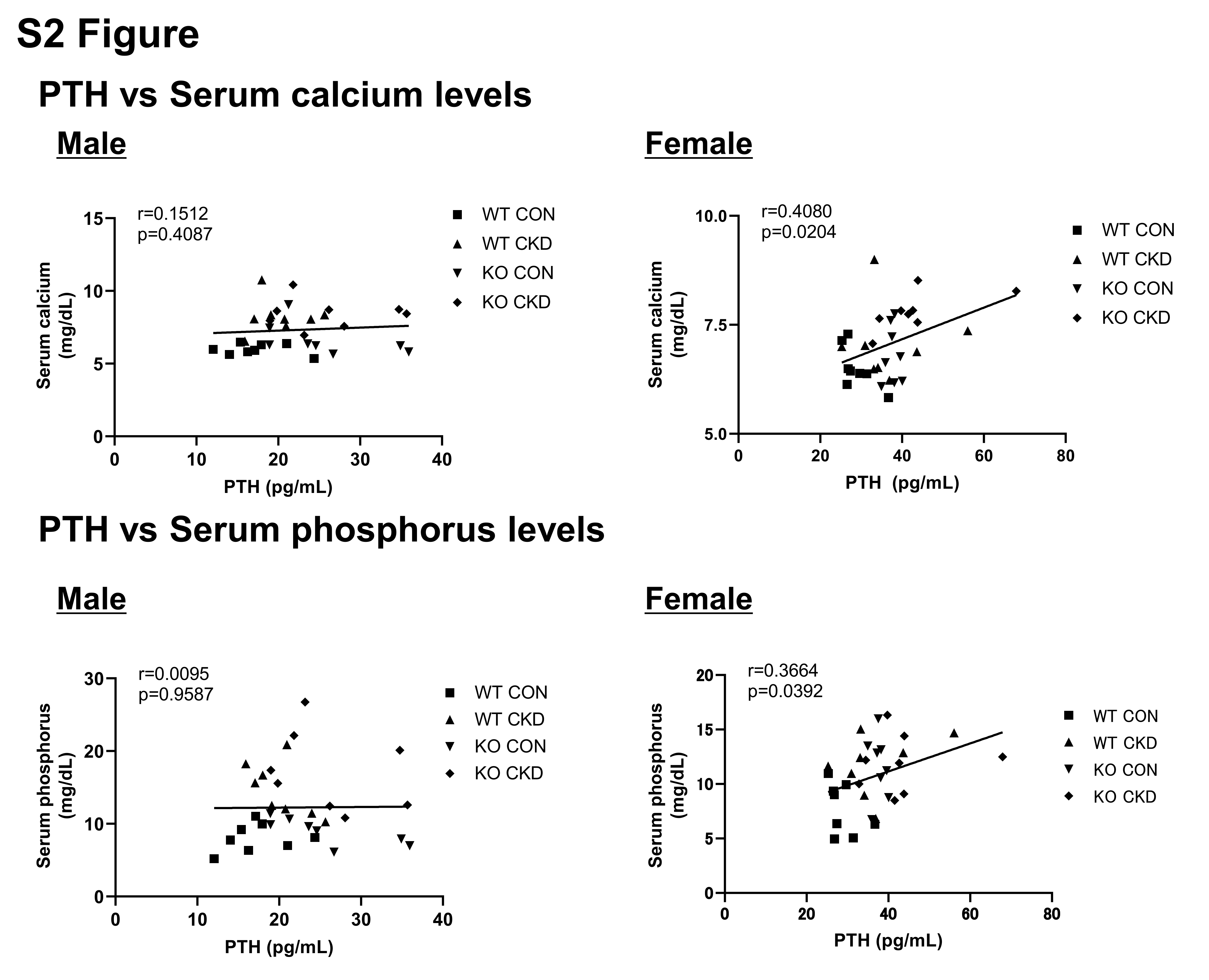

Supplement: S2 Fig — 2D scatter plots with regression line of the relationship between serum PTH levels and serum calcium (upper) and phosphorus (lower) levels in all males (left) and females (right) mice used in the present study. A simple regression analysis was performed using Spearman’s rank nonparametric correlation test. (r: Spearman’s rank correlation coefficient; p: p value). (TIF) [file pone.0311902.s002.tif]
