# Supplementary material for: Effects of plasminogen activator inhibitor-1 deficiency on bone disorders and sarcopenia caused by adenine-induced renal dysfunction in mice
Source: PLoS One. 2024 Oct 10;19(10):e0311902. doi: 10.1371/journal.pone.0311902 (PMC11469609; doi:10.1371/journal.pone.0311902)
Supplement: S3 Table — Simple regression analyses were performed between serum PTH levels and muscle mass in the whole body, muscle mass in the lower limbs, trabecular bone mineral density (BMD), the bone volume fraction (BV/TV), cortical tissue mineral density (CtTMD), cortical bone area (Ct.Ar), cortical thickness (Ct.Th) in male or female PAI-1+/+ and PAI-1-/- mice with or without adenine administration (n = 8 mice in each group). A simple regression analysis was performed with Spearman’s rank nonparametric correlation test. (r: Spearman’s rank correlation coefficient, *p<0.05, **p<0.01). (DOCX) [file pone.0311902.s005.docx]

**S3 Table.** **Simple regression analyses between serum PTH levels and muscle/bone parameters.**

|  | Male | | |  | Female | |  |
| --- | --- | --- | --- | --- | --- | --- | --- |
|  | r | *P* |  | | r | *P* | |
| Muscle mass in the whole body | -0.2152 | 0.2369 |  |  | -0.5773 | 0.0005** | |
| Muscle mass in the lower limbs | -0.1470 | 0.4221 |  |  | -0.4853 | 0.0049* | |
| Grip strength | -0.1995 | 0.2738 |  |  | -0.0909 | 0.6206 | |
| Trabecular BMD | -0.1891 | 0.2998 |  | | -0.0429 | 0.8157 | |
| BV/TV | -0.0748 | 0.6841 |  | | 0.0125 | 0.9460 | |
| CtTMD | -0.1826 | 0.3173 |  | | -0.4410 | 0.0115* | |
| Ct.Ar | -0.1789 | 0.3273 |  | | -0.6162 | 0.0002** | |
| Ct.Th | -0.2511 | 0.1657 |  | | -0.5546 | 0.0010** | |

Simple regression analyses were performed between serum PTH levels and muscle mass in the whole body, muscle mass in the lower limbs, trabecular bone mineral density (BMD), the bone volume fraction (BV/TV), cortical tissue mineral density (CtTMD), cortical bone area (Ct.Ar), cortical thickness (Ct.Th) in male or female PAI-1^+/+^ and PAI-1^-/-^ mice with or without adenine administration (n = 8 mice in each group). A simple regression analysis was performed with Spearman’s rank nonparametric correlation test. (r: Spearman’s rank correlation coefficient, **p*<0.05, ***p*<0.01)
